# Neuroanatomical correlations of visuospatial processing in primary progressive aphasia

**DOI:** 10.1093/braincomms/fcac060

**Published:** 2022-03-14

**Authors:** Boon Lead Tee, Christa Watson Pereira, Sladjana Lukic, Lynn P. Bajorek, Isabel Elaine Allen, Zachary A. Miller, Kaitlin B. Casaletto, Bruce L. Miller, Maria Luisa Gorno-Tempini

**Affiliations:** 1 Memory and Aging Center, University of California at San Francisco, San Francisco, CA, USA; 2 Department of Neurology, Dyslexia Center, University of California, San Francisco, CA, USA; 3 Global Brain Health Institute, University of California, San Francisco, CA, USA; 4 Department of Neurology, Buddhist Tzu Chi General Hospital, Hualien, Taiwan; 5 Tzu Chi University, No. 701號, Section 3, Zhongyang Rd, Hualien City, Hualien County, Taiwan 970; 6 Department of Epidemiology and Biostatistics, University of California, San Francisco, CA, USA

**Keywords:** primary progressive aphasia, visuospatial

## Abstract

Clinical phenotyping of primary progressive aphasia has largely focused on speech and language presentations, leaving other cognitive domains under-examined. This study investigated the diagnostic utility of visuospatial profiles and examined their neural basis among the three main primary progressive aphasia variants. We studied the neuropsychological performances of 118 primary progressive aphasia participants and 30 cognitively normal controls, across 11 measures of visuospatial cognition, and investigated their neural correlates via voxel-based morphometry analysis using visuospatial composite scores derived from principal component analysis. The principal component analysis identified three main factors: visuospatial-executive, visuospatial-memory and visuomotor components. Logopenic variant primary progressive aphasia performed significantly worst across all components; nonfluent/agrammatic variant primary progressive aphasia showed deficits in the visuospatial-executive and visuomotor components compared with controls; and the semantic variant primary progressive aphasia scored significantly lower than nonfluent/agrammatic variant primary progressive aphasia and control in the visuospatial-memory component. Grey matter volumes over the right parieto-occipital cortices correlated with visuospatial-executive performance; volumetric changes in the right anterior parahippocampal gyrus and amygdala were associated with visuospatial-memory function, and visuomotor composite scores correlated significantly with the grey matter volume at the right precentral gyrus. Discriminant function analysis identified three visuospatial measures: Visual Object and Space Perception and Benson figure copy and recall test, which classified 79.7% (94/118) of primary progressive aphasia into their specific variant. This study shows that each primary progressive aphasia variant also carries a distinctive visuospatial cognitive profile that corresponds with grey matter volumetric changes and in turn can be largely represented by their performance on the visuomotor, visuospatial-memory and executive functions.

## Teaser

The three canonical variants of a primary progressive aphasia (PPA) carry different visuospatial cognitive profiles. Tee *et al.* demonstrate that the distinct visuospatial cognitive phenotypes are associated with anatomical changes over the right hemisphere, albeit the structural changes of PPA are most evident over the left hemisphere.

## Graphical Abstract

**Graphical Abstract:**
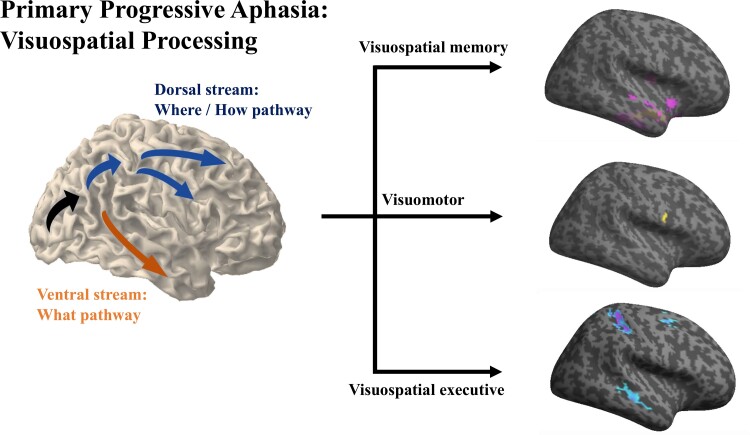

## Introduction

Primary progressive aphasia (PPA) represents a neurodegenerative syndrome that manifests predominantly with speech and language impairments.^1^ PPA is commonly classified into three main variants: the semantic variant PPA (svPPA), the nonfluent/agrammatic variant PPA (nfvPPA) and the logopenic variant PPA (lvPPA).^1^ Individuals with svPPA are generally identified by a loss of semantic knowledge, which results in difficulties with naming and single-word comprehension, while exhibiting disproportionate atrophy in the left anterior temporal lobe. Patients with nfvPPA are characterized by agrammatism and/or apraxia of speech with left frontoinsular atrophy. Individuals with lvPPA show impairments in phonology and auditory-verbal short-term memory (i.e. phonological loop) and display atrophy in the posterior portion of the left superior/middle temporal gyri into the inferior parietal lobule.^2^

Previous neurocognitive studies on PPA have largely centred on its typical neurolinguistic features. Although by criteria definition, individuals with PPA typically present with relatively isolated speech and language difficulties, few studies have highlighted the presence of non-language cognitive deficits, particularly when examined with neuropsychological tests.^3–6^ These non-language impairments are frequently related to the patients’ difficulties in manipulating verbal information. However, in some cases, these deficits also reflect changes in network-level connectivity and the disease evolution that gradually involves regions outside of the language networks. For instance, svPPA individuals exhibit deficits in verbal memory recall,^6^ but such findings are believed to be confounded by their verbal semantic functions. On the other hand, the behavioural and appetite changes that are found in svPPA,^4,5,7^ are shown to be related to the spreading of disease into orbitofrontal and right temporal areas.^8,9^ Similarly, nfvPPA individuals score lower in executive tasks,^3,6^ possibly linked to the prefrontal spread of the disease.^10^ Numerous studies also indicated that individuals with lvPPA display lower performance on memory recall.^3,6,11^ This finding may be related to deficits in verbal auditory short-term memory that interfere with learning but maybe a product of disease progression that subsequently involve the hippocampal regions or clinical heterogeneity.

Among the various non-language cognitive domains, visuospatial cognition is uniquely suited to examine the cognition patterns of PPA beyond the speech and language functions, as visuospatial tasks are relatively less dependent on verbal information. In a previous study, lvPPA individuals demonstrated worse performance across multiple visuospatial tasks,^12^ suggesting a core impairment in visuospatial processing. Conversely, nfvPPA patients showed deficits only in visuospatial tasks that relied on executive functioning but were spared in visual delayed recall.^3,12^ Finally, svPPA showed weaker performance in delayed recall of visual information but otherwise had spared visuospatial cognition.^12^ While the cognitive mechanisms of visuospatial impairment in PPA have begun to be investigated, the neuroanatomical basis leading to such visuospatial phenotypes remains to be elucidated.

The network model of visuospatial processing offers an enticing juxtaposition to the ventral and dorsal streams that have also been theorized for speech and language functioning.^13^ Generally, the ventral pathway, connects the occipitotemporal to the anterior temporal cortices and is commonly implicated in comprehension, learning and long-term memory of verbal (left hemisphere) or visual stimuli (right hemisphere). The dorsal pathway (DP) involves a distributed network connecting the temporo-occipital to frontoparietal regions and is associated with working memory, and the auditory- and visual-motor feedback loops that inform articulation and spatial navigation functions, respectively.^14–16^ Thus, the ventral pathway is recognized as the ‘what’ pathway^17–20^ and DP is termed the ‘where/how’ pathway.^16,20,21^ Examining the cognitive and anatomical mechanisms of early visuospatial deficits in PPA should provide useful information regarding the disease vulnerabilities and possibly increase diagnostic accuracy.

In this study, we examined the neural correlates of numerous visuospatial tasks among the three main PPA variants and investigated the diagnostic utility of visuospatial profiles. We hypothesized that each PPA variant would show a differential association between MRI-based anatomical damage and impairment in specific visuospatial tasks.

## Materials and methods

### Participants

A total of 148 participants were included in this study. All participants were recruited from the Memory and Aging Center at the University of California, San Francisco between the year 2002–16 and underwent comprehensive assessments that consisted of a neurological history and examination, neuropsychological testing and MRI neuroimaging. Among them, 30 participants were diagnosed as cognitively normal, and 118 participants fulfilled the PPA diagnostic criteria.^22^ The PPA participants were further classified into svPPA (*n* = 45), nfvPPA (*n* = 39) or lvPPA (*n* = 34) based on the consensus criteria established in 2011.^1^ After reviewing the clinical history, neuropsychological data and MRI neuroimaging, diagnoses were established among a multidisciplinary team that consisted of one or two behavioural neurologists, a clinical neuropsychologist, and/or a genetic counsellor and a speech-language pathologist. We excluded participants that had a mini-mental state examination (MMSE; Folstein *et al.*^23^) score < 8 or a Clinical Dementia Rating (CDR) scale > 2 and had not undergone MRI imaging within 6 months of their neuropsychological testing. For participants with longitudinal follow-up, we included data from the initial evaluation that consist of both cognitive and imaging data. Written informed consent for inclusion in this research was obtained from all participants or their medical proxies. This study received ethical approval from the University of California, San Francisco (UCSF) Committee on Human Research and was conducted in accordance with the Declaration of Helsinki.

The demographics, neuropsychological, speech and language data for all groups are reported in Table 1 and 2. There were no significant differences among the three PPA variants and the healthy control groups in terms of education level, sex and handedness. The nfvPPA participants were slightly older in age of onset [*F*(2,115) = 7.44, *P* = 0.001] and at examination [*F*(3,144) = 3.93, *P* = 0.010] when compared with the other PPA variants but comparable in the time point of assessment since disease onset [*F*(2,115) = 3.01, *P* = 0.053)]. As expected, MMSE was significantly lower and CDR scores higher in the PPA groups when compared with the healthy controls [*F*(3,144) = 22.52, *P* < 0.0001]. The lvPPA participants also performed lower on the MMSE score in comparison with the other two PPA variants, and the nfvPPA participants had a lower CDR sum of boxes score [*F*(2,115) = 7.58, *P* = 0.001] but a comparable proportion of individuals with lower CDR grades compared with lvPPA and svPPA groups (*P* = 0.115).

**Table 1:** Demographic characteristics and neuropsychological test scores of the study participants (*n* = 148).

	svPPA (*n* = 45)	nfvPPA (*n* = 39)	lvPPA (*n* = 34)	Control (*n* = 30)	*P*-value
**Demographic**					
** Age of onset**	58.11 (6.55)^a^	63.95 (7.87)^a,b^	58.82 (7.79)^b^	—	0.001
** Years from onset to examination**	5.00 (2.98)	3.85 (1.93)	4.12 (2.07)	—	0.077
** Age at examination**	63.11 (5.42)^a^	67.79 (7.59)^a,b^	62.94 (8.14)^b^	63.60 (8.0)	0.010
**Sex**					
** Female**	23	27	19	13	0.307
** Male**	22	13	15	17	
**Education (years)**	16.38 (3.18)	15.83 (2.73)	16.38 (3.12)	17.28 (2.27)	0.296
**Handedness**					
** Right**	40	36	29	26	0.434
** Left**	3	4	5	2	
** Ambidextrous**	2	0	0	2	

Note: values are mean (standard deviation).

lvPPA, logopenic variant primary progressive aphasia; nfvPPA, nonfluent/agrammatic variant primary progressive aphasia; svPPA, semantic variant primary progressive aphasia

**Table 2:** The neuropsychological test scores of the study participants (*n* = 148)

	svPPA (*n* = 45)	nfvPPA (*n* = 39)	lvPPA (*n* = 34)	Control (*n* = 30)	*P*-value
**Global cognition and function**					
** MMSE**	25.80 (3.14)^c,f^	25.84 (3.94)^b,e^	20.47 (6.72)^b,c,d^	28.87 (1.14)^d,e,f^	<0.0001
** CDR sum of boxes**	3.52 (2.39)^a^	1.76 (1.46)^a,b^	3.32 (2.65)^b^	–	0.001
** CDR**					
** 0**	4^a,f^	10^a,b,e^	3^b,d^	30^d,e,f^	<0.0001
** 0.5**	27	24	22	0	
** 1.0**	13	5	7	0	
** 2.0**	1	0	2	0	
**Working memory/executive function**					
** Digit span forward** ^g^	6.5 (1.38)^a,c^	4.88 (1.12)^a,e^	4.14 (0.96) ^c,d^	6.88 (1.33) ^d,e^	<0.0001
** Digit span backward** ^h^	5 (1.38)^a,c^	3.57 (1.12)^a,e^	2.85 (1.08)^c,d^	5.43 (1.14)^d,e^	<0.0001
** Stroop colour naming** ^i^	71.61 (2.83)^a,c,f^	43.87 (2.93)^a,e^	46.46 (3.50)^c,d^	88.0 (2.75)^d,e,f^	<0.0001
** Stroop colour interference** ^j^	42.68 (2.07)^a,c,f^	26.21 (2.02)^a,e^	19.07 (2.32)^c,d^	51.27(2.07)^d,e,f^	<0.0001
** Phonemic fluency (D-letter)** ^k^	8.5 (4.40)^f^	6.81 (4.98)^e^	6.82 (4.51)^d^	17.04 (3.56)^d,e,f^	<0.0001
** Semantic fluency (animal)** ^l^	9.11 (5.48)^f^	11.84 (7.66)^e^	8.52 (5.19)^d^	23.38 (5.96)^d,e,f^	<0.0001
**Verbal episodic memory**					
** CVLT-SF 1–4 trials (0–40)** ^m^	16.86 (6.87)^a,f^	23.33 (5.61)^a,b,e^	14.97 (7.42)^b,d^	29.63 (3.08)^d,e,f^	<0.0001
** CVLT-SF 30 s** ^m^	3.16 (2.47)^a,f^	6.44 (1.72)^a,b,e^	3.61 (2.77)^b,d^	8.21 (1.03)^d,e,f^	<0.0001
** CVLT-SF 10 min** ^m^	2.30 (2.43)^a,f^	6.00 (2.20)^a,b^	2.81 (2.93)^b,d^	7.74 (1.33)^d,f^	<0.0001
**Speech and language**					
** Boston naming test (0–15)** ^n^	5.51 (3.64)^a,c,f^	12.18 (2.82)^a,b,e^	9.00 (4.04)^b,c,d^	14.41 (0.83)^d,e,f^	<0.0001
** Peabody Picture Vocabulary Test (0–16)** ^o^	8.27 (4.55)^a,c,d^	14.44 (2.05)^a^	13.96 (1.95)^c^	15.65(0.71)^d^	<0.0001
** Repetition—short form (0–5)** ^o^	3.85 (1.04)^a,c,f^	2.63 (1.63)^a,e^	2.08 (1.22)^c,d^	4.75 (0.52)^d,e,f^	<0.0001
** Verbal agility (0–5)** ^p^	5.26 (1.29)^a,c^	2.41 (1.48)^a,b,e^	3.58 (1.47)^b,c,d^	5.13 (2.01)^d,e^	<0.0001
** Syntax comprehension-short form (0–5)** ^q^	4.51 (0.72)	4.16 (1.05)	3.76 (1.05)	4.24 (1.64)	0.074
** WAB fluency rating (0–10)** ^r^	8.85 (0.89)^a^	6.65 (2.60)^a,b^	8.23 (1.67)^b^	–	<0.0001
** AOS severity rating (0–7)** ^s^	0.13 (0.57)^a^	2.64 (1.95)^a,b^	0.55 (1.24)^b^	–	<0.0001
** Dysarthria severity rating (0–7)** ^t^	0.13 (0.09)^a^	2.19 (2.40)^a,b^	0 (0)^b^	–	<0.0001
** WAB sequential command (0–80)** ^u^	76.40 (8.38)^a,c^	73.94 (8.22)^a,b^	67.59 (13.62)^b,c^	–	<0.0001
**Syntax comprehension**					
** UCSF syntax comprehension (%)** ^v^	96 (7.12)	93 (7.71)	90.07 (8.02)	–	0.069
** Cycle syntax comprehension (%)** ^w^	93.71 (6.46)^a,c^	80.29 (12.94)^a^	68.46 (18.60)^c^	–	<0.0001
** Syntax production (0–16)** ^x^	15.63 (0.81)^c^	14.60 (2.46)	13.33 (2.87)^c^	–	0.034
** Inflection morphology(0–80)** ^y^	63.69 (7.72)	65.73 (7.43)	58.13 (8.22)		0.091
** WAB repetition (0–100)** ^z^	91.46 (10.29)^a,c^	81.55 (20.10)^a,b^	71.29 (13.14)^b,c^	–	<0.0001

Note: Values are mean (standard deviation).

AOS, apraxia of speech; CDR, Clinical Dementia Rating; CVLT-SF, short form California Verbal Learning Test; lvPPA, logopenic variant primary progressive aphasia; MMSE, mini-mental state examination; nfvPPA, nonfluent/agrammatic variant primary progressive aphasia; svPPA, semantic variant primary progressive aphasia; WAB, Western Aphasia Battery.

^a^ Significant between nfvPPA and svPPA;

^b^ Significant between nfvPPA and lvPPA;

^c^ Significant between svPPA and lvPPA;

^d^ Significant between control and lvPPA;

^e^ Significant between control and nfvPPA;

^f^ Significant between control and svPPA;

^g^ lvPPA (*n* = 21), nfvPPA (*n* = 24), svPPA (*n* = 30), Control (*n* = 24);

^h^ lvPPA (*n* = 34), nfvPPA (*n* = 37), svPPA (*n* = 44), Control (*n* = 30);

^i^ lvPPA (*n* = 24), nfvPPA (*n* = 30), svPPA (*n* = 36), Control (*n* = 28);

^j^ lvPPA (*n* = 26), nfvPPA (*n* = 32), svPPA (*n* = 41), Control (*n* = 29);

^k^ lvPPA (*n* = 34), nfvPPA (*n* = 37), svPPA (*n* = 44), Control (*n* = 28);

^l^ lvPPA (*n* = 33), nfvPPA (*n* = 37), svPPA (*n* = 45), Control (*n* = 29);

^m^ lvPPA (*n* = 31), nfvPPA (*n* = 36), svPPA (*n* = 44), Control (*n* = 19);

^n^ lvPPA (*n* = 34), nfvPPA (*n* = 39), svPPA (*n* = 45), Control (*n* = 29);

^o^ lvPPA (*n* = 25), nfvPPA (*n* = 35), svPPA (*n* = 39), Control (*n* = 28);

^p^ lvPPA (*n* = 26), nfvPPA (*n* = 37), svPPA (*n* = 39), Control (*n* = 24);

^q^ lvPPA (*n* = 25), nfvPPA (*n* = 38), svPPA (*n* = 39), Control (*n* = 25);

^r^ lvPPA (*n* = 31), nfvPPA (*n* = 34), svPPA (*n* = 40);

^s^ lvPPA (*n* = 29), nfvPPA (*n* = 33), svPPA (*n* = 39);

^t^ lvPPA (*n* = 28), nfvPPA (*n* = 32), svPPA (*n* = 39);

^u^ lvPPA (*n* = 29), nfvPPA (*n* = 34), svPPA (*n* = 40);

^v^ lvPPA (*n* = 14), nfvPPA (*n* = 20), svPPA (*n* = 24);

^w^ lvPPA (*n* = 13), nfvPPA (*n* = 14), svPPA (*n* = 14);

^x^ lvPPA (*n* = 9), nfvPPA (*n* = 10), svPPA (*n* = 16);

^y^ lvPPA (*n* = 8), nfvPPA (*n* = 15), svPPA (*n* = 16);

^z^ lvPPA (*n* = 31), nfvPPA (*n* = 33), svPPA (*n* = 41).

### Neuropsychological assessment

The neuropsychological testing was conducted by neuropsychology fellows or research staff under the supervision of board-certified neuropsychologists, the CDR assessment was completed by registered nurses, and the speech and language assessment data were collected by board-certified speech-language pathologists. All participants completed a neuropsychological battery as illustrated in Kramer *et al.*^24^ In particular, the neuropsychological battery consisted of an MMSE for global cognition assessment,^23^ the short form California Verbal Learning Test (CVLT-SF) for verbal episodic memory evaluation,^25,26^ the forward and backward digit span length for working memory, the Stroop colour naming/interference test and category fluency test for executive function assessment,^24^ and the short version of motor speech,^27^ repetition^28^ and syntax comprehension tests^29,30^ for speech and language assessment. PPA participants are further evaluated by a detailed speech and language battery. The comprehensive speech and language battery consisted of a fluency rating, sequential commands and repetition tests from the Western Aphasia Battery,^28^ and motor speech tests as illustrated in Ogar *et al.*^27^ with apraxia of speech and a dysarthria severity rating score,^31^ the 15 items Boston Naming Test (BNT),^32^ the Peabody Picture Vocabulary Test (PPVT)^33^ and syntax comprehension tests^29,30^ to reflect the motor speech, semantic and syntactic functions.

For the visuospatial battery, we collected 11 visuospatial measures: the number location test from Visual Object and Space Perception battery^34^ to assess visuospatial location; the abbreviated Beery visual-motor integration (VMI) test,^12^ the Benson figure copy test^12,35^ and the Wechsler Memory Scale (WMS) Visual Reproduction I test^36^ to reflect the visuomotor integration ability; the Benson figure delayed recall^12,35^ and the WMS Visual Reproduction II test^36^ as measures of visuospatial-memory function; the forward and backward spatial span length to represent spatial attention and spatial working memory function, respectively; the Wechsler Adult Intelligence Scale (WAIS) Block Design^36^ to evaluate visuospatial construction skill; the modified Trails B test^24,37^ to characterize visuospatial sequencing and switching function; and the Delis–Kaplan Executive Function System (DKEFS) Design Fluency filled dots^38^ to indicate spatial fluency ability.

### MRI image acquisition and pre-processing

T_1_-weighted MRI images were collected via 1.5 T and 3 T MRI scanners with a standard quadrature head coil (eight channels) at UCSF MAC. The T_1_-weighted images were acquired using an MPRAGE sequence with the following parameters: 160 sagittal slices, repetition time = 2300 ms, echo time = 2.98 ms, inversion time = 900 ms, flip angle 9°, field of view = 256 mm^3^, matrix size = 256 × 240, voxel in-plane size = 1.0 × 1.0 mm^2^; slice thickness = 1 mm.

The neuroimaging data of PPA participants were pre-processed using the Computational Anatomy Toolbox (CAT12; http://dbm.neuro.uni-jena.de/cat) within Statistical Parametric Mapping software (SPM12; http://www.fil.ion.ucl.ac.uk/spm/software/spm12) running on the Matlab 2018b programme (http://www.mathworks.com). The T_1_-weighted images were spatially aligned with the Montreal Neurological Institute (MNI) space then segmented into grey matter (GM), white matter (WM) and CSF. The GM and WM tissue were further normalized using DARTEL (http://www.fil.ion.ucl.ac.uk/spm/software/spm12). The normalized and modulated GM segments were smoothed by 8 mm full-width at half-maximum Gaussian Kernel.

### Statistical analysis

#### Behavioural data

Group differences in the demographic variables, neuropsychological, visuospatial, speech and language measures were examined using Pearson’s *χ*^2^ test for categorical variables, ANOVA with Bonferroni *post hoc* analysis for continuous variables compared across the three PPA and control groups, and Student’s *t*-test for subgroup analysis of two sample continuous variables. An alpha level <0.05 was adopted to determine statistical significance in the group differences comparison.

To compute the visuospatial composite scores that best represent the visuospatial performances in this PPA cohort, we applied principal component analysis (PCA) using the visuospatial measures to identify variables measuring similar latent cognitive performance. There were <4.1% missing data for each visuospatial measure, and the missing visuospatial data were replaced by the means for each specific group to facilitate the PCA. The visuospatial measures were first standardized via *Z*-score conversion using the mean and standard deviation of all participants. Visuospatial measures then underwent PCA analysis with oblique rotation (Direct Oblimin rotation) with Kaiser normalization.^39^ Based on the eigenvalue, three components were extracted. Each factor with a loading >0.5 was considered to contribute heavily to the particular component. To account for the language influences in our visuospatial imaging and discriminant analysis, a similar PCA method was also applied for language data that was collected in both PPA and control groups (i.e. PPVT, BNT, short version of motor speech, repetition and syntax comprehension test) to compute language composite scores. Using general linear model, the visuospatial measures were adjusted with age on examination, MMSE score and language composite scores as covariates. We then adopted stepwise discriminant function analysis to identify the covariate-adjusted visuospatial measures that contribute most in discriminating the three PPA variants and delineate the predictability of visuospatial measures in the classification of PPA variants. Statistical analyses were conducted using IBM SPSS Statistics 26.0 (https://www.ibm.com/support/pages/downloading-ibm-spss-statistics-26).

#### Imaging data

To identify the GM atrophy regions that correlate with the visuospatial composite values using the structural MRI images of 118 PPA participants, voxel-based morphometry (VBM) analysis with a multiple linear regression was adopted. Age, handedness, total intracranial volume, gender, language composite scores and PPA diagnosis were included as covariates in each of the regression analyses. For the neural correlate analyses reported in this study, the clusters and voxel threshold were set at *P* < 0.05 and family-wise error (FWE) corrected. The VBM analysis was conducted using Statistical Parametric Mapping software (SPM12) running on the Matlab 2017 programme.

### Data availability

The clinical and neuroimaging data used in this study are available from the corresponding author upon reasonable request. Given the sensitive nature of participants’ data, our ethics protocol does not permit open data sharing at this stage.

## Results

### Neuropsychological, speech and language performances

On neuropsychological testing, lvPPA and nfvPPA performed worse on working memory and executive tasks such as digit span forward [*F*(3,98) = 26.42, *P* < 0.0001] and backwards [*F*(3,141) = 34.83, *P* < 0.0001], Stroop colour naming [*F*(3,114) = 47.76, *P* < 0.0001] and interference tasks [*F*(3,124) = 42.58, *P* < 0.0001] (Table 2). For verbal fluency tests, the PPA participants generated fewer accurate verbal responses than the control participants, both in phonemic [*F*(3,139) = 36.26, *P* < 0.0001] and semantic fluency tasks [*F*(3,140) = 39.63, *P* < 0.0001]. On the CVLT-SF, svPPA and lvPPA learned and recalled significantly fewer words when compared with the controls and nfvPPA individuals [CVLT-SF 1–4 Trials: *F*(3,126) = 28.43, *P* < 0.0001; CVLT-SF 30 s: *F*(3,126) = 32.79, *P* < 0.0001; CVLT-SF 10 min: *F*(3,126) = 33.87, *P* < 0.0001].

The speech and language data showed that the PPA groups scored significant lower on the BNT as compared with the control group, particularly for svPPA and lvPPA groups [*F*(3,143)  = 55.85, *P* < 0.0001]. In addition to naming difficulties, svPPA participants also demonstrated significantly lower PPVT scores [*F*(3,129) = 45.19, *P* < 0.0001]. The Western Aphasia Battery (WAB) fluency was significantly reduced in the nfvPPA [*F*(2,102) = 13.94, *P* < 0.0001], and they also exhibited significantly higher speech apraxia and dysarthria rating scores [speech apraxia rating: *F*(2,98) = 34.12, *P* < 0.0001; dysarthria rating: *F*(2,96) = 24.33, *P* < 0.0001]. On the WAB sequential command and the WAB repetition test, the scores were significantly decreased in the order of svPPA, nfvPPA and lvPPA [WAB sequential command: *F*(2,100) = 6.60, *P* = 0.002; WAB repetition: *F*(2,102) = 16.48, *P* < 0.0001]. In terms of syntax comprehension and production tests, the nfvPPA group performed poorly for syntax comprehension and the lvPPA group scored lower in both syntax tasks when compared with the svPPA group [UCSF syntax production: *F*(2,55) = 2.81, *P* = 0.069; Cycle syntax production: *F*(2,38) = 11.93, *P* < 0.0001; syntax production: *F*(2,32) = 3.75, *P* = 0.034].

### Visuospatial measures and composite scores

The performances of PPA and control groups on the 11 visuospatial measures are depicted in Table 3. Participants with lvPPA scored significantly lower on all 11 visuospatial measures when compared with the control participants. Similarly, nfvPPA participants scored significantly lower than the control groups in most visuospatial measures, with the exception of the number location test, modified Trails B (accuracy/completion time), Benson figure copy and recall and Beery VMI measures. In contrast, svPPA participants only performed worse than the control participants on Benson figure recall, WMS Visual Reproduction I and II, DKEFS Design Fluency and WAIS Block Design tasks. Direct comparisons among the three PPA variants, lvPPA scored the lowest among all the visuospatial measures. When compared to svPPA, individuals with nfvPPA also scored significantly lower in modified Trails B, Spatial span backward, and WAIS Block Design tasks; but they showed significantly better performances on Benson figure recall, WMS Visual Reproduction II tests. Individuals with svPPA scored significantly lower in tasks that are dependent on Benson figure recall, WMS Visual Reproduction II tests, and they were relatively intact in their other visuospatial measures.

**Table 3:** The visuospatial measures and composite scores of the study participants (*n* = 148)

	svPPA(*n* = 45)	nfvPPA(*n* = 39)	lvPPA(*n* = 34)	Control(*n* = 30)	ANOVA	
					*P*-value	*F*
**Modified trail**	0.411 (0.188)^a,c,f^	0.289 (0.214)^a,b,e^	0.148 (0.157)^b,c,d^	0.552 (0.227)^d,e,f^	<0.0001	25.12
**Spatial backward**	5.111 (1.133)^a,c^	4.256 (1.292)^a,e^	3.567 (1.146)^c,d^	5.333 (0.802)^d,e^	<0.0001	18.13
**Spatial forward**	5.400 (1.053)^c^	4.846 (1.288)^e^	4.233 (1.272)^c,d^	5.700 (0.915)^d,e^	<0.0001	10.76
**Block design**	33.578 (10.903)^a,c,f^	25.846 (14.243)^a,e^	19.849 (10.629)^c,d^	41.167 (11.102)^d,e,f^	<0.0001	20.14
**Design fluency**	7.798 (3.265)^f^	6.700 (2.733)^e^	6.375 (3.373)^d^	10.067 (2.532)^d,e,f^	<0.0001	9.74
**Number location**	9.230 (1.240)^c^	8.864 (1.341)	7.935 (2.282)^c,d^	9.200 (1.064)^d^	0.001	5.43
**Benson recall**	7.133 (4.230)^a,f^	10.692 (3.450)^a,b^	6.606 (3.284)^b,d^	12.567 (2.738)^d,f^	<0.0001	22.36
**Visual Reproduction II**	18.222 (19.368)^a,f^	37.487 (26.640)^a,b,e^	15.032 (17.733)^b,d^	61.900 (22.480)^d,e,f^	<0.0001	32.42
**Visual Reproduction I**	63.200 (19.697)^c,f^	65.513 (22.416)^b,e^	47.935 (21.310)^b,c,d^	84.200 (13.095)^d,e,f^	<0.0001	18.04
**Benson Copy**	15.467 (1.198)^c^	14.897 (2.036)	13.676 (3.906)^c,d^	15.367 (1.189)^d^	0.005	4.51
**Beery VMI**	13.822 (2.081)^c^	12.103 (3.939)	10.441 (4.150)^c,d^	13.733 (2.132)^d^	<0.0001	8.88
**Principal component analysis: visuospatial measures**						
**Factor 1: executive**	0.451 (0.649)^a,c^	−0.346 (0.932)^a,e^	−0.820 (0.932)^d,c^	0.702 (0.727)^d,e^	<0.0001	25.85
**Factor 2: memory**	0.426 (0.890)^a,f^	−0.209 (0.802)^a,b,e^	0.628 (0.720)^b,d^	−1.080 (0.650)^d,e,f^	<0.0001	31.59
**Factor 3: motor**	−0.338 (0.502)^c^	0.055 (0.998)	0.558 (1.513)^c,d^	−0.197 (0.435)^d^	<0.0001	6.27

Notes: Values are represented as mean score (standard deviation) for visuospatial measures; *Z*-score (standard deviation) for the composite scores; visuospatial measures and composite scores were analysed across the three PPA and control groups using ANOVA analysis with *F-* and *P*-value presented (all df1 = 3, df2 = 144).

lvPPA, logopenic variant primary progressive aphasia; nfvPPA, nonfluent/agrammatic variant primary progressive aphasia; svPPA, semantic variant primary progressive aphasia.

^a^ Significant between nfvPPA and svPPA;

^b^ Significant between nfvPPA and lvPPA;

^c^ Significant between svPPA and lvPPA;

^d^ Significant between control and lvPPA;

^e^ Significant between control and nfvPPA;

^f^ Significant between control and svPPA.

**Figure 1:**
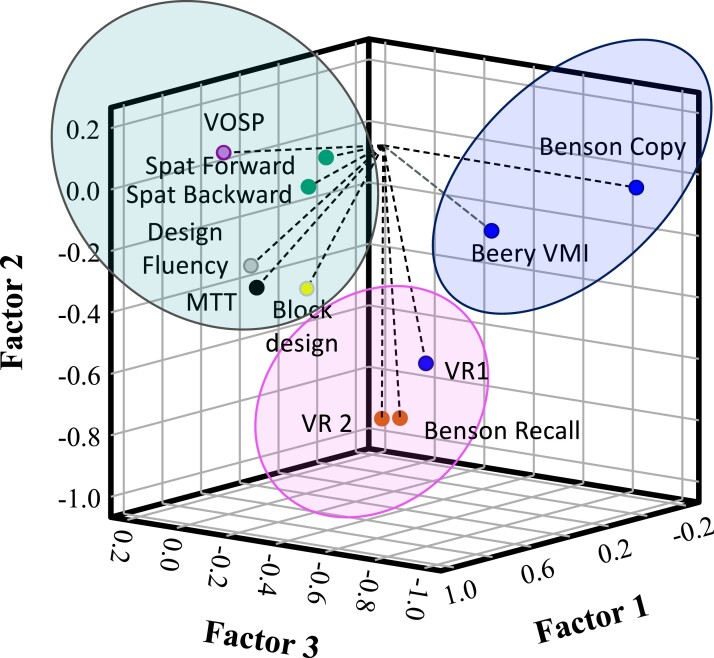
**PCA of the 11 visuospatial measures.** This figure displays the vector projections of each visuospatial measure among the derived three principle components. Note that the green, red and blue rings encircling the visuospatial measures with factor loading >0.5 for the principal components 1, 2 and 3, respectively. The labels of the visuospatial measures were coloured based on the visuospatial functions: purple, number location test for visuospatial localization function; blue, Beery VMI, Benson figure copy and Visual Reproduction I for visuomotor integration ability; vermillion, Benson figure delayed recall and Visual Reproduction II test for visuospatial-memory function; bluish green, forward and backward spatial span length for spatial attention and spatial working memory function; yellow, Block Design for visuospatial construction skill; black, modified Trails B test for visuospatial sequencing and switching function; grey, Design Fluency for spatial fluency ability.

The 11 visuospatial measures were subjected to an oblimin-rotated with Kaiser normalization PCA, and it produced three visuospatial factors (VSP-Factor) with eigenvalues of 5.61, 1.16 and 0.89 (Fig. 1). These factors accounted for 69.7% of the variance of the visuospatial scores produced by the PPA and control participants (VSP-Factor 1 = 51.01%, VSP-Factor 2 = 10.57% and VSP-Factor 3 = 8.09%).

The factor loadings of each visuospatial measure are listed in Fig. 1 and Table 4. VSP-Factor 1 is heavily loaded with visuospatial measures that also have a high executive function load, such as the modified Trails B test, the forward and backward spatial span length, the DKEFS Design Fluency filled dots and the WAIS Block Design. These measures, respectively, primarily represent the visuospatial sequencing and switching function, spatial attention and spatial working memory function, spatial fluency ability and visuospatial problem-solving. As these functions tapped into different levels of visuospatial-executive functions, VSP-Factor 1 will be referred to as the visuospatial-executive component. Conversely, VSP-Factor 2 was mainly dependent on the performance on the Benson figure delayed recall and the WMS Visual Reproduction I and II test, whereas VSP-Factor 3 was heavily weighted by the scores on the Beery VMI and the Benson figure copy test. The Benson figure delayed recall and the WMS Visual Reproduction II test are measures of visuospatial memory. On the other hand, the Beery VMI, the Benson figure copy and the WMS Visual Reproduction I test reflect visuomotor integration ability. Owing to the joint structure of the WMS Visual Reproduction I and II tests, scores across these two metrics naturally show high correlations (Pearson correlation coefficient *r* = 0.76) that tilted the WMS Visual Reproduction I test to represent Factor 2. Taking this into consideration, VSP-Factor 2 can be interpreted as the visuospatial-memory component, and VSP-Factor 3 is the visuospatial motor component.

**Table 4:** Principal component analysis based on the *Z*-scores of the 11 visuospatial measures (*n* = 148)

	Principal component analysis		
	1	2	3
**Spatial backward**	**0.805**	−0.375	−0.535
**Block design**	**0.781**	−0.647	−0.476
**Modified trail**	**0.741**	−0.619	−0.310
**Spatial forward**	**0.723**	−0.270	−0.515
**Design fluency**	**0.683**	−0.537	−0.248
**Number location**	**0.632**	−0.202	−0.168
**Visual Reproduction II**	0.446	−**0.907**	−0.319
**Benson recall**	0.307	−**0.862**	0.238
**Visual Reproduction I**	0.589	−**0.815**	0.583
**Benson Copy**	0.315	−0.284	−**0.931**
**Beery VMI**	0.606	−0.470	−**0.811**

Note: The bold values represent the most heavily weighted loading values of each visuospatial measure.

The lvPPA and nfvPPA groups performed significantly worse than svPPA and control groups on the visuospatial-executive composite score [*F*(3,145) = 25.85, *P* < 0.0001]. In contrast, the nfvPPA and the control group scored significantly higher than the lvPPA and svPPA groups on the visuospatial-memory component [*F*(3,145) = 31.59, *P* < 0.0001]. As for the composite score representing the visuospatial motor component, the lvPPA participants were found to score significantly lower in comparison with the svPPA and control groups [*F*(3,145)  = 6.27, *P* < 0.0001].

### Language composite scores

Two language components (L-Factor) with eigenvalues of 1.87 and 1.67 were identified via PCA approach using the five language measures that were collected in all PPA and control groups (Supplementary Table 1). These factors accounted for 70.79% of the variance produced by the PPA and control participants (L-Factor 1 = 37.42% and L-Factor 2 = 33.37%). L-Factor 1 is heavily loaded with language measures that target motor speech, repetition and syntax comprehension functions; L-Factor 2 is heavily weighted with language measures that reflect naming and semantic functions. As expected, nfvPPA and lvPPA scored lower than svPPA and controls in L-Factor 1. Compared with controls and nfvPPA, svPPA and lvPPA showed significantly lower scores in L-Factor 2 (Supplementary Table 2).

### Neuroanatomical correlation of the visuospatial composite measures

**Figure 2:**
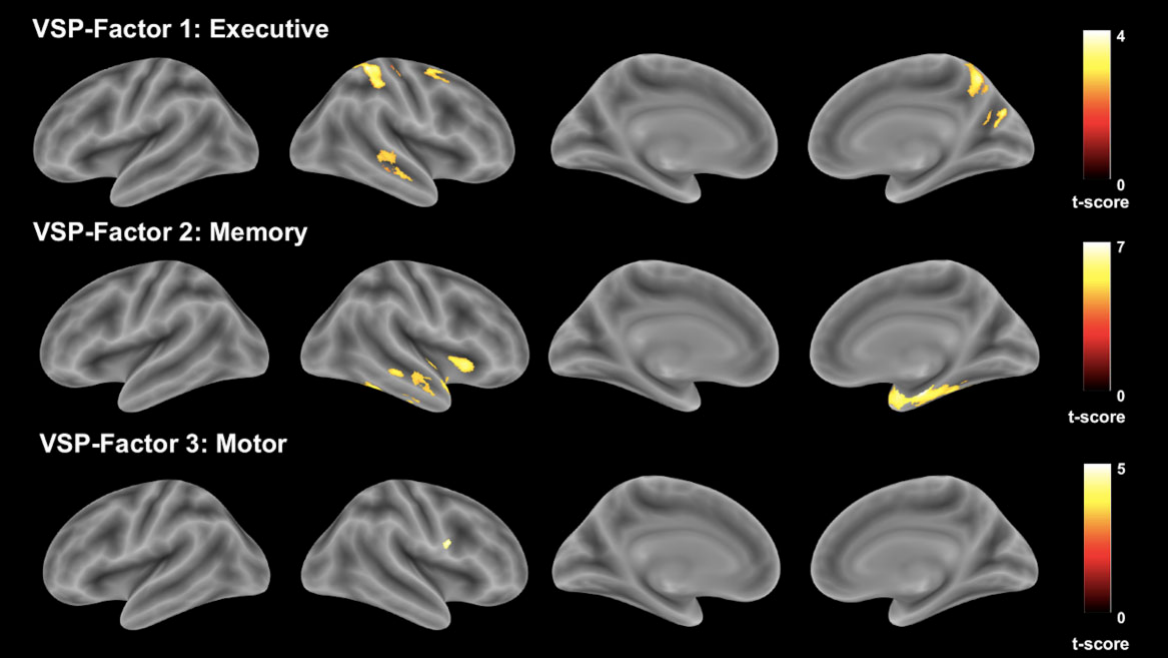
**Neuroanatomical correlation of visuospatial factor components.** The figure shows the neuroanatomical correlation analysis of the visuospatial composite scores with the GM volumetric changes via the VBM method, adjusting for age at examination, sex, handedness, total intracranial matter volume, PPA diagnosis, and language composite scores. The clusters and voxel threshold was set at *P* < 0.05 and FWE corrected. Note that the neural correlates with visuospatial-executive composite score was significant at cluster threshold but did not survive the voxel-wise threshold of *P* < 0.05 after FWE statistical correction. Maps are superimposed on an inflated standard brain in the MNI space. Hot colour bars represent *t*-score of the correlations.

To investigate the neuroanatomical basis of visuospatial performances in PPA, performances on the three visuospatial composite scores were correlated with the GM regions using VBM analysis (Fig. 2 and Table 5). The visuospatial-memory composite score was specifically correlated with the right amygdala, hippocampus, anterior and middle temporal cortices. Performance on the visuospatial motor composite score showed unique correlations with the voxels in the right precentral gyrus. The visuospatial-executive composite score was correlated with the GM volume in right superior parietal lobule, right cuneal and precuneal cortices, postcentral gyrus, right middle frontal and posterior middle temporal gyri.

**Table 5:** Neuroanatomical correlates of the visuospatial factor components

Regions	Extent	*t*-value	MNI coordinates		
			*x*	*y*	*z*
**Factor 1: executive**					
**Right superior parietal lobule**	2203	4.83	26	−44	57
**Right precuneus cortex**		4.51	8	−60	52
**Right postcentral gyrus**		4.44	26	−39	45
**Right cuneal cortex**	466	4.39	15	−78	22
**Right precuneus cortex**		3.80	15	−64	16
		3.53	24	−70	22
**Right middle frontal gyrus**	462	3.80	26	−3	50
**Right posterior middle temporal gyrus**	726	3.72	59	−29	−8
**Factor 2: memory**					
**Right amygdala**	7827	7.54	22	−8	−15
**Right parahippocampal gyrus**		7.48	22	−16	−24
**Right amygdala**		7.13	28	3	−22
**Right insula**	1492	6.61	33	18	0
**Right temporal pole**		5.67	56	16	−21
**Right insula**		5.58	46	10	−9
**Right middle temporal gyrus**	204	6.00	58	−32	−9
**Right superior temporal gyrus**		5.49	64	−26	−4
**Right inferior temporal gyrus**	96	5.51	54	−21	−28
**Factor 3: motor**					
**Right precentral gyrus**	228	5.96	56	6	22

### Stepwise discriminant analysis of visuospatial measures

Stepwise discriminant analysis identified three of the 11 visuospatial measures as variables with the highest discriminant power (Supplementary Table 3). Listed in descending order, the measures are the visual object and space perception, Benson figure recall and Benson figure copy scores (*χ*^2^(12) = 63.38, *F* = 5.078, *P* < 0.0001). With these three visuospatial measures, 94 of 118 PPA participants (79.7%) were accurately classified (Table 6). Similarly, 78.0% of the cases were correctly classified with the cross-validated method using these three visuospatial measures. The svPPA and nfvPPA groups showed high congruency between the visuospatial measures predicted diagnosis and the criteria diagnosis (91.1% and 92.3%, respectively). Among the 34 lvPPA participants, only 17 participants were accurately predicted and all but one of the remaining cases were misclassified as nfvPPA. When comparing the demographic features and cognitive measures between the accurately classified lvPPA and the misclassified-as-nfvPPA lvPPA, the former group was found to have significantly lower MMSE score (*t* = −7.475, *P* = 0.006), higher CDR sum of box score (*t* = 1.866, *P* = 0.019) and CDR global score (*P* = 0.098) with lower performance in BNT (*t* = −6.101, *P* = 0.018), apraxia of speech severity rating (*t* = 2.655, *P* < 0.0001) and syntax comprehension test (*t* = −1.705, *P* = 0.016) (Supplementary Table 4).

**Table 6:** Stepwise discriminant function analysis of PPA participants with number location test, Benson recall and Benson copy tasks

Classification results				
Criteria diagnosis	Predicted PPA variants			
	svPPA	nfvPPA	lvPPA	Total
svPPA	**41 (91.1)**	2 (4.4)	2 (4.4)	45
nfvPPA	0 (0)	**36** (**92.3)**	3 (7.7)	39
lvPPA	1 (2.9)	16 (47.1)	**17** (**50)**	34

Notes: number of participants (percentage). The bold values represent the number of participants in which the predicted diagnoses using the three visuospatial measures matched the clinical diagnoses.

lvPPA, logopenic variant primary progressive aphasia; nfvPPA, nonfluent/agrammatic variant primary progressive aphasia; PC, principal component; svPPA, semantic variant primary progressive aphasia.

## Discussion

In this study, we describe distinct visuospatial cognitive profiles among the three PPA variants and examined the neural basis of visuospatial processing in PPA using GM volume and visuospatial composite scores derived from the PCA. The predictability of the visuospatial measures was studied via discriminant function analysis to identify visuospatial measures that best discriminate the three PPA variants.

Analysis of the visuospatial battery that consisted of 11 visuospatial measures supports the hypothesis that each PPA variant carries a distinctive pattern of visuospatial deficits. Specifically, svPPA participants exhibited deficits in visuospatial memory but their other visuospatial functions were intact. Conversely, the nfvPPA participants displayed relative preservation of their visuospatial-memory ability, despite evident impairment in the other executive and motor visuospatial measures. Among the three PPA variants, individuals with lvPPA showed the lowest visuospatial performances across all evaluated visuospatial measures. Previous studies have found that lvPPA individuals have lower performance on any tasks heavily dependent on visuospatial localization,^3,12^ visuospatial short-term memory,^6,12,40,41^ visuospatial attention,^12,42,43^ visuospatial working memory^12,40,43,44^ and visuomotor integration functions.^12^ Ramanan *et al.* and Watson *et al.*’s studies also demonstrated that nfvPPA and lvPPA differ in their visuospatial short-term memory performances. The visuospatial cognitive profiles shown in this study are parallel the visuospatial cognitive profiles found in our PPA cohort. Our findings demonstrate that although the PPA syndrome predominantly affects the speech and language networks, it also impacts the less investigated visuospatial cognitive functions. Interestingly, Weintraub *et al.*^45^ previously reported that clinically diagnosed PPA patients scored significantly better than patients with amnestic Alzheimer’s disease and were comparable with controls when tasked to perform the delayed recall of simple geometric designs. Compared with patients with amnestic Alzheimer’s disease, Mesulam *et al.*^46^ also found that PPA patients with Alzheimer’s biomarkers or pathology have relatively preserved episodic memory in recalling the picture of common objects. We speculate that the variable interpretations of visuospatial-memory function in PPA could have stemmed from the visuospatial complexity of stimuli used (e.g. simple geometric design versus complex figure), the distributions of PPA variants in the PPA cohort and the nature of stimuli (e.g. delayed recall of common objects may also be dependent on semantic memory function).

The PCA outcomes underscored the pivotal role of the visuospatial-executive, memory and motor functions in portraying the visuospatial profiles of the PPA individuals. Factor 1 is derived from a collection of visuospatial tasks targeting various aspects of visuospatial-executive functions including visuospatial sequencing and switching function, spatial attention and working memory function, spatial generativity ability and visuospatial construction skill. Based on this, we can infer that these visuospatial functions share common neural networks and/or neuroanatomical regions that are affected by the different disease pathophysiology found in PPA individuals. Factors 2 and 3 comprised of tasks reflecting visuospatial memory and visuomotor integration skills, respectively, with the exception of the visual reproduction I task, which is commonly regarded as an indicator of visuomotor integration function. We speculate that the performance on the visual reproduction I was derived as a component of Factor 2, and not Factor 3, given its strong correlation with the performance on the visual reproduction II task.

The discriminant analysis identified visual object and space perception, Benson figure recall and Benson figure copy scores as the three visuospatial measures most pivotal in classifying PPA variants. Coincidentally, these three visuospatial measures each belong to a different visuospatial PCA component. Thus, instead of collecting all 11 visuospatial measures, administering three non-language dependent visuospatial tasks that target visuomotor, visuospatial memory and executive functions can be a time-efficient alternative approach in representing the visuospatial profile of PPA variants. The predictability of PPA variants with the visuospatial measures reached 79.7%, even without relying on the speech and language measures. Therefore, in addition to idiosyncratic language features, the three PPA variants also carry distinctive visuospatial cognitive profiles that potentially provides reference to the classification of PPA variants. This is especially true for individuals with svPPA, nfvPPA and lvPPA based on the congruency rate between the predicted and criteria diagnosis. Interestingly, lvPPA individuals with milder global cognitive impairments display visuospatial profiles which resembles those of nfvPPA individuals. Leyton *et al.*^47^ demonstrated presence of clinical heterogeneity in lvPPA patients; thus, we speculate that the variations in visuospatial cognitive profiles may be related to the clinical heterogeneity of lvPPA.

In general, the neural basis of the visuospatial composite scores corresponds well with the neural framework for visuospatial processing. More specifically, the visuospatial-executive composite score correlates with the GM volumetric changes over the right posterior parietal, right visual association cortices and right middle frontal gyrus. Right posterior parietal and visual association cortices are key regions of the occipito-parietal circuit for visuospatial processing. This circuit is commonly regarded as the anatomical precedent circuit of the three pathways in the dorsal visuospatial processing streams: parieto-prefrontal, parieto-premotor and parieto-medial temporal pathways. The occipito-parietal circuit is responsible for transforming the retinotopic represented visual information into egocentric frame of reference.^14–16^ Right posterior parietal and prefrontal cortices have also been reported to be critical for regulating visuospatial attention, visuospatial working memory and spatial localization.^44,48–51^ The visuospatial-executive composite score is a derivational product of the modified Trail B test, spatial span length, WAIS Block Design, visual object and space perception and DKEFS Design Fluency tests, and these tests are primarily dependent on spatial attention, localization and working memory functions. It is thus unsurprising that the visuospatial-executive composite score in PPA individuals showed a correlation with the GM volumes over the right posterior parietal, right visual association and right frontal cortices. On the other hand, the ventral stream network of visuospatial processing is most commonly implicated in the identification, learning and episodic memory of visual stimuli. Based on its projections, it can be divided into six distinct pathways: occipitotemporo-neostriatal, occipitotemporo-amygdaloid, occipitotemporo-ventral striatum, occipitotemporo-medial temporal, occipitotemporo-orbitofrontal and occipitotemporo-ventrolateral prefrontal pathways.^52^ Our study showed that the visuospatial-memory composite performance in PPA individuals is strongly associated with the ventral stream network that projects towards the anterior and medial temporal cortices, primarily involving the occipitotemporo-amygdaloid and occipitotemporo-medial temporal pathways. Generally, the occipitotemporo-medial temporal supports the formation of short-term and long-term visual memory and the occipitotemporo-amygdaloid pathway is related to the effective processing of visual stimuli.^14–16,52^ Hence, the visuospatial-memory composite score, which is mainly composed of visuospatial measures that heavily depend on visual short-term memory, is associated with the volumetric changes over these regions. Similarly, the visuospatial motor composite score, which is composed of visuospatial tasks measuring visuomotor abilities, exhibited neuroanatomical correlation with the GM volume over the right precentral gyrus in PPA individuals. We believe that this region is critical for the parietal–premotor pathway of the dorsal stream network that is known to support visually guided actions.^14–16^

Interestingly, the visuospatial cognitive profile and its neural basis of the three PPA variants displayed mirroring neural signatures to the language pathways, albeit involving the contralateral/right hemisphere. Individuals with svPPA that are recognized for their damage over the anterior temporal lobes and ventral WM tracts showed lower performance in visuospatial measures that associate with the ventral stream of the visuospatial neural framework; whereas nfvPPA that are known for left frontoinsular atrophy and altered connectivity over the frontoparietal WM tracts scored lower on visuospatial measures that rely on the dorsal stream visuospatial network. Based on the longitudinal VBM study on the atrophy pattern of lvPPA individuals,^53^ we speculate that visuospatial performance in lvPPA individuals is related to the disease involvement over the right occipital-parietal circuit at the early stage of the disease. The disease pathology eventually spreads towards the anterior and medial temporal cortices and results in visual memory deficits. These findings support the notion that the progressive neural network degeneration reported in PPA variants, although predominantly, does not exclusively involve the speech and language network only. Studying these non-language cognitive deficits may help inform the disease evolution and potentially serve as markers for disease severity.

This study has several limitations that warrant consideration. First, our study showed that based on the visuospatial phenotypes alone, only 79.7% of the PPA participants showed congruent PPA variants diagnosis as the consensus criteria. This level of compatibility is arguably insufficient to serve as sole markers for differentiating PPA variants. This may be attributed to the fact that most visuospatial tasks are dependent on verbal/written instructions and motor production abilities and that there is the lack of optimal measures to statistically account for the disease severity of PPA individuals. Second, previous cortical thickness studies of lvPPA participants showed that the spatial span length was related to the left superior parietal or bilateral temporo-parieto-occipital brain regions.^43,44^ In comparison, our neural correlate results showed relatively more right hemispheric involvements. This is probably because PPA is a clinical syndrome that typically shows more left-hemispheric atrophy, the limited right hemispheric volumetric changes may have restricted adequate variance to support strong anatomical correlation findings over the right hemisphere. Thus, in this study, we computed language composite scores to statistically adjust for possible neural correlates contributed by language measures. Finally, the PPA and control groups were not equally matched across all demographic variables. In comparison with the svPPA and lvPPA group, the nfvPPA group displayed a higher mean age of onset and a lower CDR sum of box score. The discrepancy in the CDR score may indicate variability in severity across the PPA variants. Nevertheless, the time point of assessment since disease onset did not vary across the PPA variants and the visuospatial profiles noted in our study were in line with the previous literature, suggesting the limited impact of the heterogeneity in the CDR score.^12,40,42–44^ To minimize any possible confounding effect of age and clinical severity, the GM volumetric analysis in this study was adjusted for age at examination.

In summary, this study describes the distinctive visuospatial cognitive profile of the three PPA variants, outlines the neural basis underlying these visuospatial deficit patterns and examines the predictability of the visuospatial measures in classifying the PPA variants. Overall, our findings highlight the relevance of non-language presentations in PPA individuals and demonstrate the added benefit of incorporating visuospatial measures in the standard evaluation of PPA presentations.

## Supplementary Material

fcac060_Supplementary_Data

## Abbreviations

CDR =: Clinical Dementia Rating
CVLT-SF =: short form California Verbal Learning Test
DP =: dorsal pathway
lvPPA =: logopenic variant primary progressive aphasia
MMSE =: mini-mental state examination
nfvPPA =: nonfluent/agrammatic variant primary progressive aphasia
PCA =: principal component analysis
PPA =: primary progressive aphasia
PPVT =: Peabody Picture Vocabulary Test
svPPA =: semantic variant PPA
VBM =: voxel-based morphometry
