# Supplementary material for: Neuroanatomical correlations of visuospatial processing in primary progressive aphasia
Source: Brain Commun. 2022 Mar 14;4(2):fcac060. doi: 10.1093/braincomms/fcac060 (PMC8977647; doi:10.1093/braincomms/fcac060)
Supplement: fcac060_Supplementary_Data [file fcac060_supplementary_data.docx]

Supplementary table 1. Principal component analysis based on the five speech and language measures. (n=148)

| Principal Component Analysis | | |
| --- | --- | --- |
|  | 1 | 2 |
| Verbal agility | **0.834** | -0.270 |
| Repetition - short form | **0.753** | 0.121 |
| Syntax comprehension – short form | **0.709** | 0.074 |
| Boston Naming Test | -0.062 | **0.914** |
| Peabody Picture Vocabulary Test | 0.080 | **0.913** |

Supplementary table 2. The language composite scores of the study participants (n=148).

|  | svPPA  (n=45) | nfvPPA  (n=39) | lvPPA  (n=34) | Control  (n=30) | p-value |
| --- | --- | --- | --- | --- | --- |
| Principal Component Analysis: Speech and Language measures | | | | | |
| Factor 1 | 0.442 (0.739)^a,c^ | -0.603(0.852)^a,e^ | -0.483 (0.782)^c,d^ | 0.670 (1.032)^d,e^ | <0.0001 |
| Factor 2 | -1.015 (0.970)^a,c,f^ | 0.141(0.712)^a,b^ | -0.740 (0.633)^b,c^ | 0.400( 0.120)^f^ | <0.0001 |

Notes: Values are mean (standard deviation).

lvPPA = logopenic variant Primary Progressive Aphasia; nfvPPA = nonfluent/agrammatic variant Primary Progressive Aphasia svPPA = semantic variant Primary Progressive Aphasia

^a^ Significant between nfvPPA and svPPA; ^b^ Significant between nfvPPA and lvPPA; ^c^ Significant between svPPA and lvPPA; ^d^ Significant between control and lvPPA; ^e^ Significant between control and nfvPPA; ^f^ Significant between control and svPPA

Supplementary Table 3. Stepwise discriminant analysis of the visuospatial measures.

| **Step** | **Visuospatial measures** | **Lambda** | **Exact F** | |  | |
| --- | --- | --- | --- | --- | --- | --- |
|  |  |  | **Statistic** | **df1** | **df2** | **p-value** |
| **1** | Benson Copy | 0.522 | 52.551 | 2 | 115 | <0.0001 |
| **2** | Number location test | 0.233 | 61.184 | 4 | 228 | <0.0001 |
| **3** | Benson Recall | 0.208 | 44.876 | 6 | 226 | <0.0001 |

Supplementary table 4. The demographic characteristics and cognitive scores of lvPPA patients that are accurately classified (n=17) and misclassified as nfvPPA (n=16).

|  | Accurately-classified lvPPA  (n=17) | Misclassified-as-nfvPPA lvPPA  (n=16) | p-value | |
| --- | --- | --- | --- | --- |
| Demographic |  |  |  | |
| Age at onset | 60.00 (8.60) | 57.50 (7.17) | 0.214 | |
| Years from onset to exam | 4.35 (2.40) | 3.88 (1.78) | 0.157 | |
| Age at exam | 64.35 (9.43) | 61.38 (6.78) | 0.066 | |
| Sex  Female  Male | 10  7 | 8  8 | 0.611 | |
| Education (years) | 15.41 (3.12) | 17.31 (3.11) | 0.646 | |
| Handedness  Right  Left  Ambidextrous | 15  2  0 | 13  3  0 | 0.576 | |
| Global Cognition and Function |  |  |  | |
| MMSE | 15.18 (5.10) | 25.81 (2.81) | 0.006 | |
| CDR Sum of Boxes | 4.18 (3.15) | 2.53 (1.77) | 0.019 | |
| CDR  0  0.5  1.0  2.0 | 0  10  5  2 | 3  11  2  0 | 0.098 | |
| Principal Component Analysis: Visuospatial measures | | | |  |
| Factor 1: Executive | -1.22 (0.90) | -0.45 (0.83) | 0.924 | |
| Factor 2: Memory | 0.97 (0.40) | 0.26 (0.84) | 0.009 | |
| Factor 3: Motor | 0.88 (1.99) | 0.27 (0.75) | 0.009 | |
| Working memory/ Executive function | | | |  |
| Digit Forward | 3.38 (0.52) | 4.58 (0.90) | 0.305 | |
| Digit Backward | 2.35 (1.06) | 3.38 (0.89) | 0.680 | |
| Stroop Color Naming | 35.63 (19.29) | 51.00 (13.52) | 0.269 | |
| Stroop Color Interference |  |  |  | |
| Phonemic Fluency (D-letter) | 4.41 (3.12) | 8.81 (4.23) | 0.495 | |
| Semantic Fluency (Animal) | 5.19 (4.02) | 11.44 (4.18) | 0.793 | |
| Verbal episodic memory | | | |  |
| CVLT-SF 1-4 Trials (0-40) | 11.00 (5.89) | 18.93 (7.05) | 0.557 | |
| CVLT-SF 30 seconds | 2.00 (2.20) | 5.27 (2.40) | 0.819 | |
| CVLT-SF 10 mins | 1.27 (1.83) | 4.53 (2.95) | 0.067 | |
| Speech and Language | | | |  |
| Boston Naming Test (0-15) | 6.29 (3.42) | 12.13 (1.89) | 0.018 | |
| Peabody Picture Vocabulary Test (0-16) | 13.35 (1.73) | 14.69 (1.45) | 0.625 | |
| Repetition - short form (0-5) | 1.44 (0.527) | 2.40 (1.40) | 0.063 | |
| Verbal agility (0-5) | 2.89 (1.54) | 3.81 (1.28) | 0.607 | |
| Syntax comprehension – short form (0-5) | 3.46 (0.95) | 4.00 (0.73) | 0.016 | |
| WAB Fluency Rating (0-10) | 7.72 (1.87) | 8.67 (1.40) | 0.308 | |
| AOS Severity Rating (0-7) | 1.14 (1.61) | 0 (0) | <0.0001 | |
| Dysarthria Severity Rating (0-7) | 0 (0) | 0 (0) | - | |
| WAB Sequential Command (0-80) | 7.73 (1.87) | 8.67 (1.40) | 0.308 | |
| WAB Repetition (0-100) | 63.33 (9.71) | 78.51 (11.98) | 0.256 | |

Notes: Values are mean (standard deviation) for continuous variables and number of participants for categorical variables.

^a^ Significant between nfvPPA and svPPA; ^b^ Significant between nfvPPA and lvPPA; ^c^ Significant between svPPA and lvPPA;

^d^ Significant between control and lvPPA; ^e^ Significant between control and nfvPPA; ^f^ Significant between control and svPPA
